# Expression and anti-apoptotic function of TRAF4 in human breast cancer MCF-7 cells

**DOI:** 10.3892/ol.2013.1703

**Published:** 2013-11-25

**Authors:** XIAOLI ZHANG, ZHIFENG WEN, XIAOYI MI

**Affiliations:** 1 Department of Pathology, The First Affiliated Hospital and College of Basic Medical Sciences of China Medical University, Shenyang, Liaoning 110001, P.R. China; 2 Department of Assisted Reproduction, Shenyang Women’s and Children’s Hospital, Shenyang, Liaoning 110001, P.R. China; 3 Department of Neurosurgery, The First Affiliated Hospital, China Medical University, Shenyang, Liaoning 110001, P.R. China

**Keywords:** TRAF4, apoptosis, breast cancer, siRNA

## Abstract

Tumor necrosis factor (TNF) receptor-associated factor 4 (TRAF4) was initially identified as a gene amplified and overexpressed in breast carcinoma. The present study investigated the expression and anti-apoptotic function of TRAF4 in human breast cancer MCF-7 cells. TRAF4 was found to be localized in the cytoplasm and nuclei of MCF-7 cells by immunofluorescence staining and western blotting. The expression of TRAF4 in normal MCF-10A breast cells was found to be lower than in MCF-7 and MDA-MB-231 breast cancer cells. Following TNF-α treatment, TRAF4 depletion by siRNA in the MCF-7 cells was observed to suppress cell proliferation and the nuclear expression of nuclear factor κB was significantly reduced. The percentage of early apoptotic cells in the MCF-7 cells was augmented upon TRAF4-knockdown, and an increase in G_1_ phase cells and a decrease in S phase cells was detected. These results indicate that TRAF4 has anti-apoptotic effects on apoptosis induced by TNF-α in MCF-7 cells.

## Introduction

Tumor necrosis factor (TNF) receptor-associated factors (TRAFs) have emerged as the major signal transducers for the TNF receptor (TNFR) superfamily and the interleukin-1 receptor/Toll-like receptor superfamily. TRAFs interact directly or indirectly with TNFRs to regulate signaling events, including the activation of nuclear factor κB (NF-κB) and c-Jun N-terminal kinase (JNK) (1,2). TRAFs collectively play important roles, including actions in adaptive and innate immunity, embryonic development, the stress response and bone metabolism. These functions are mediated by TRAFs through the induction of cell survival, proliferation, differentiation and death (3–8).

TRAF4 shares most sequence similarity with *Drosophila* TRAF1, an adapter molecule critical for JNK activation and eye development in the fruit fly (9). Originally identified as a protein localized in the nuclei of breast carcinoma cells, TRAF4 has also been previously detected in the cytoplasm (10–12). Although *in vivo* studies have shown that TRAF4 is involved in important biological functions (4,8,10), how it functions at the molecular level remains elusive.

Our previous study detected the expression of TRAF4 in normal and cancerous breast tissues. The results demonstrated that the TRAF4 nuclei positive rate in normal breast tissue is significantly higher than in non-invasive (P<0.01) and invasive (P<0.05) ductal carcinomas (13).

The present study investigated the expression and location of TRAF4 in breast cancer cells and the biological function of TRAF4 in MCF-7 cells.

## Materials and methods

### Cell culture and treatment

The human normal MCF-10A breast cell line and the human MCF-7 and MDA-MB-231 breast cancer cell lines were obtained from the American Type Culture Collection (Manassas, VA, USA). Normal MCF-10A breast cells were cultured in Dulbecco’s modified Eagle’s medium (DMEM)/F12 (1:1) supplemented with 5% equine serum, 10 μg/ml insulin and 20 ng/ml epidermal growth factor. MDA-MB-231 breast cancer cells were cultured in L15 supplemented with 10% fetal bovine serum (FBS) and 100 units penicillin-streptomycin. MCF-7 was routinely cultured in DMEM supplemented with 10% FBS and 100 units penicillin-streptomycin. All the cells were cultured at 37°C with 5% CO_2_ in a humidified incubator.

### Western blot analysis

Samples (50 μg) were separated by SDS-PAGE and transferred to a polyvinylidene fluoride membrane. The membrane was blocked with 5% skimmed milk and incubated overnight at 4°C with the primary antibodies. Next, the membranes were incubated in the secondary antibodies for 2 h at room temperature with slight agitation. The ECL western blotting detection system (Amersham Pharmacia Biotech, Amersham, UK) was used for their detection.

### Antibodies

The membranes for western blotting were incubated with mouse anti-human monoclonal antibodies against TRAF4 (1:1,000; BD Biosciences, Franklin Lakes, NJ, USA), NF-κB p65 (1:1,000; Beyotime Institute of Biotechnology, Haimen, China), lamin B1 (1:500; Santa Cruz Biotechnology, Inc., Santa Cruz, CA, USA) and β-actin (1:1,000; Santa Cruz Biotechnology Inc.), followed by horseradish peroxidase-conjugated secondary antibody (Santa Cruz Biotechnology Inc.).

### Immunofluorescence staining

Cells grown on glass coverslips were fixed with ice-cold 4% paraformaldehyde for 15 min at −20°C, followed by permeabilization with 0.2% Triton X-100. The cells were incubated with anti-TRAF4 (1:100; Santa Cruz Biotechnology) antibodies at 4°C overnight, followed by incubation with a secondary antibody conjugated to rhodamine. The nuclei were counterstained with 4′6-diamidino-2-phenylindole and observations were performed with a confocal microscope (Leica SP1 and SP2 UV; Leica, Mannheim, Germany).

### Plasmid and transfection

TRAF4 siRNA sequences (Santa Cruz Biotechnology Inc.) were transfected with HiPerFect transfection reagent (Qiagen, Hilden, Germany) into cells to accomplish the transient transfection according to the manufacturer’s instructions. Control siRNA-A (Santa Cruz Biotechnology Inc.) was used as a negative control.

### Flow cytometry

Flow cytometry was performed using an apoptosis detection kit (KeyGen Biotech, Nanjing, China), according to the manufacturer’s instructions, on a BD FACSCalibur™ (BD Biosciences) flow cytometer. The percentages of cells in the various cell cycle phases were determined using the FACSCalibur flow cytometer with CellQuest 3.0 software (BD Biosciences). All experiments were performed in triplicate.

### Cell proliferation assay

The MCF-7 cells (1×10^3^) were grown in 96-well plates. Following various treatments, the cells were further incubated with MTT (0.5 mg/ml) at 37°C for 4 h, followed by the addition of 150 μl DMSO. The absorbance values were measured at 550 nm using a microplate reader (Bio-Rad, Hercules, CA, USA).

### Statistic analysis

All values are expressed as the mean ± SD. Student’s t-test was used to analyze all results using the statistical software package, SPSS 13.0 (SPSS, Inc., Chicago, IL, USA). For all the tests, P<0.05 was considered to indicate a statistically significant difference.

## Results

### Expression and location of TRAF4 in breast cancer cells

**Figure 1:**
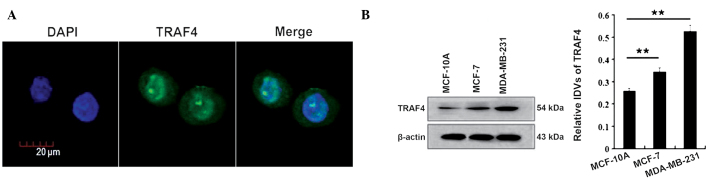
Expression and location of TRAF4 in breast cancer cells. (A) Results of immunofluorescence staining showing TRAF4 (green fluorescence) localized in the cytoplasm and nuclei (scale bar, 20 μm). (B) MCF-10A, MCF-7 and MDA-MB-231 cells were lysed and the lysates were subjected to western blot analyses of TRAF4 and β-actin. Results showed that the total expression of TRAF4 in the MCF-10A cells was lower than in the MCF-7 and MDA-MB-231 cells (^**^P=0.002 and P=0.001, respectively). TRAF4, tumor necrosis factor receptor-associated factor 4; IDV, integated optical density value.

Firstly, the cellular localization of TRAF4 was confirmed in the breast cancer MCF-7 cells through immunofluorescence staining. The results showed that TRAF4 was localized in the cytoplasm and nuclei of the MCF-7 cells, with its nuclear expression stronger than its cytoplasmic expression (Fig. 1A).

Next, the expression of TRAF4 was examined in normal MCF-10A breast cells and the estrogen receptor-positive and -negative breast cancer cell lines, MCF-7 and MDA-MB-231, respectively, by western blotting. TRAF4 was found to be expressed in all the cells, while the total expression of TRAF4 was lower in the MCF-10A cells than in the MCF-7 and MDA-MB-231 cells (P=0.002 and P=0.001, respectively; Fig. 1B).

### TRAF4 suppresses the activation of NF-κB in MCF-7 cells

**Figure 2:**
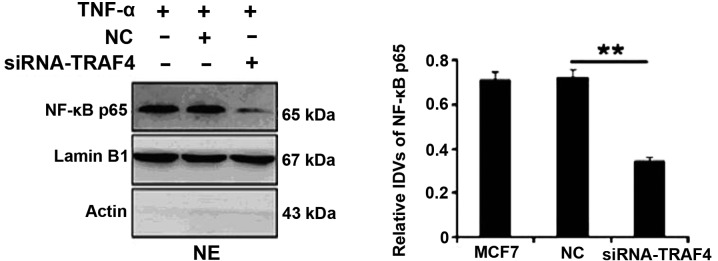
TRAF4 suppresses the activation of NF-κB in MCF-7 Cells. The MCF-7 cells were lysed and the lysates were subjected to western blot analyses of NF-κB, β-actin and lamin B1. Following TNF-α treatment (10 ng/ml; 15 min), the nuclear expression of NF-κB was significantly downregulated when TRAF4 was knocked down in the MCF-7 cells (^**^P=0.002). TRAF4, tumor necrosis factor receptor-associated factor 4; NF-κB, nuclear factor κB.

The nuclear expression of NF-κB was examined in the estrogen receptor-positive MCF-7 breast cancer cell line by western blotting. Following TNF-α treatment, TRAF4 depletion by siRNA in the MCF-7 cells significantly suppressed the nuclear expression of NF-κB (P=0.002; Fig. 2). However, no significant differences were identified in the MCF-10A cells (data not shown).

### TRAF4 may promote cell proliferation and suppress cell apoptosis in MCF-7 cells

**Figure 3:**
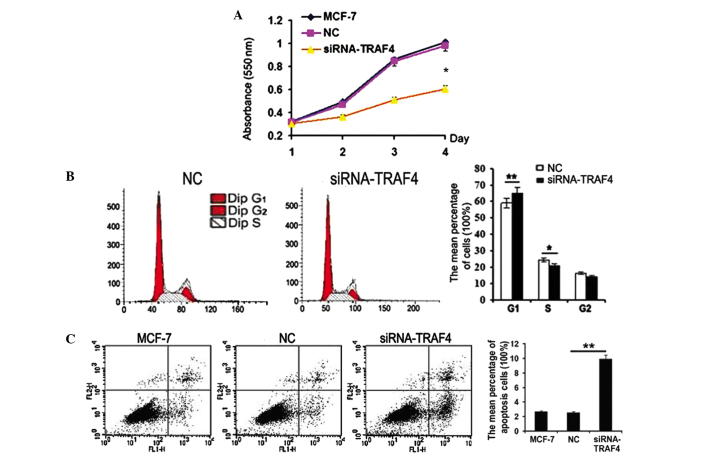
Biological function of TRAF4 in MCF-7 cells. (A) MTT results showing that cell proliferation was significantly suppressed following the knockdown of TRAF4 (^*^P=0.02). Results of flow cytometry showed that TRAF4 ablation resulted in (B) significant increase of G_1_ phase cells (^**^P=0.009) and reduction of S phase cells (^*^P=0.04) and (C) significant increase in the early apoptotic cells (^**^P=0.001). All comparisons were made between the groups of MCF-7 cells or cells transfected with negative control alone. TRAF4, tumor necrosis factor receptor-associated factor 4.

The biological function of TRAF4 was examined in the MCF-7 cells. When TRAF4 was inhibited by siRNA in the MCF-7 cells, the cell proliferation was effectively suppressed compared with the negative control (P=0.02; Fig. 3A). An increase in G_1_ phase cells (P=0.009) and a decrease in S phase cells (P=0.04) was detected when TRAF4 was knocked down (Fig. 3B). In addition, TRAF4 depletion by siRNA in the MCF-7 cells was found to markedly promote early apoptosis (P=0.001; Fig. 3C).

## Discussion

Camilleri-Broët *et al* previously demonstrated that TRAF4 overexpression is a common characteristic of human carcinomas, including lung cancer and breast, ovary, prostatic and pancreatic adenocarcinomas (14). The study indicated that one of the mechanisms responsible for TRAF4 protein overexpression in human cancer was TRAF4 gene amplification. TRAF4 is located in a region of amplification that is devoid of known oncogenes on chromosome 17q11.2, and is commonly overexpressed in cancer. The results of the present study showed that, *in vitro*, TRAF4 exhibits higher expression in breast cancer cells than in normal breast cells. In addition, TRAF4 expression in the estrogen receptor-positive breast cancer cell lines was higher than in the estrogen receptor-negative breast cancer cell lines. This is consistent with the expression of TRAF4 in breast cancer tissues reported in our previous study (13). The results of the present study in breast cancer cells and human breast tissues may indicate that TRAF4 has an important role in breast cancer.

Numerous TRAF family members negatively regulate apoptotic pathways by increasing the expression of genes that promote cell survival (15,16). Several previous studies have hypothesized that TRAF4 may also be involved in apoptosis. However, depending on the study, the role of TRAF4 in apoptosis is controversial. On the one hand, Sax *et al* previously demonstrated that TRAF4 may play a role in p53-mediated proapoptotic signaling in response to cellular stress (17). Furthermore, TRAF4 has been previously shown to suppress the ability of the common neurotrophin receptor, p75NTR, dimers to block cell death induced by p75NTR monomers, also indicating a proapoptotic role for TRAF4 (18). On the other hand, Fleckenstein *et al* hypothesized an anti-apoptotic function for TRAF4 when the study found that the anti-Fas antibody, CH-11, induces apoptosis in HEK293 cells, but not when these cells are stably transfected with TRAF4 (19). Although seemingly paradoxical, these results may all be correct depending on the cells examined. In the current study, following TNF-α treatment in breast cancer MCF-7 cells, the expression of TRAF4 suppressed the activation of NF-κB and promoted early cell apoptosis. In addition, it was demonstrated that TRAF4 ablation resulted in a significant increase in G_1_ phase cells and a reduction in S phase cells. These results indicate that TRAF4 may promote the activation of NF-κB induced by TNF-α in MCF-7 cells. Future studies must clarify the roles of TRAF4 in apoptotic reactions and may contribute to the development of a new strategy against breast cancer.
